# Expression of BCL2L12and LIPOCALIN 2 in Adult Patient with Acute Myeloid Leukemia and Correlation with Clinical Response

**DOI:** 10.31557/APJCP.2021.22.7.2267

**Published:** 2021-07

**Authors:** Lucie G khozam, Reham A.A Elshimy, Roxan E Shafik

**Affiliations:** *Department of Clinical and Chemical Pathology, National Cancer Institute, Cairo University, Egypt. *

**Keywords:** Acute myeloid leukemia, bone marrow, LCN, BCL2L12

## Abstract

**Background:**

Acute myeloid leukemia (AML) is a malignancy arising within the bone marrow (BM), in which leukemic cells proliferate uncontrollably in association with a disruption of normal hematopoiesis.Aim of the work to evaluate the expression of LCN and BCL2L2, in newly diagnosed bone marrow samples from adult with AML and to correlate their expression levels with clinical and Laboratory data of the patients especially that known to have a prognostic feature.

**Methods:**

This study was carried out on 87 consecutive newly diagnosed adult AML patients of which 75 are evaluated for both LCN, BCL2L12 (All 87 are evaluated for LCN). In addition, 20 donors of matched age and sex healthy individuals from donors for bone marrow transplantation were included as a control group.

**Results:**

No statistical significant correlation was found between LCN over-expression and the control group , there was no statistical significance between its expression and age, sex, hepatomegally, splenomegally and lymphadenopathy, also there was no statistical significance regarding peripheral blood and bone marrow findings, immunophenotyping, cytogenetics or molecular findings and MRD at day 15.No statistical significance was found between BCL2L12 expression and the control group again there was no statistical significance between its expression and age, sex, hepatomegally, splenomegally and lymphadenopathy, also there was no statistical significance regarding peripheral blood and bone marrow findings, immunophenotyping, cytogenetics or molecular findings and MRD at day 15.

**Conclusion:**

LCN and BCL2L12 were found to be expressed with non significant difference in AML as in normal subjects; however studies on large number of cases are needed to confirm our finding. The role of LCN and BCL2L12 need to be verified by further large-scale sample and further studies.

## Introduction

The most common acute leukemia in adults is the acute myeloid leukemia (AML). The pathophysiology of the disease associates with cytogenetic abnormalities, gene mutations and aberrant gene expressions. At the molecular level, the disease manifests as changes in both epigenetic and genetic signatures. At the clinical level, two aspects of AML should be taken into account. First, the molecular changes occurring in the disease are important prognostic and predictive markers of AML. Second, use of novel therapies targeting these molecular changes. Currently, cytogenetic abnormalities and molecular alterations are the common biomarkers for the prognosis and choice of treatment for AML (Pourrajab et al., 2020). 

The Lipocalin (NGAL) (LCN2) complex is found in blood tumor cells from patients with ALL, AML and CLL types of leukemia. Over expression of free NGAL is observed in blood cells from patients with all types of leukemia. NGAL does not appear to influence the balance between survival and death of bone marrow stem and progenitor CD34+ cells, mature granulocytes, and T and B lymphocytes. Whereas it blocks the maturation of lineage-committed myeloid cells into mature erythrocytes and monocytes (Wang et al., 2019). 

BCL2L12 Bcl-2, like protein 12, is a protein that in humans is encoded by the BCL2L12 gene. The protein encoded by this gene belongs to the Bcl-2 protein family. BCL2L12 expression is upregulated in most human glioblastomas. Expression of BCL2L12 results in resistance to apoptosis. BCL2L12 directly neutralizes caspase-7 (CASP7) and indirectly neutralizes caspase-3 (CASP3) by an indirect mechanism. Both caspase enzymes are known to play essential roles in the execution phase of apoptosis (Chia-Hua, 2012). In our study we aimed at evaluating the expression of LCN and BCL2L2, in newly diagnosed bone marrow samples from adult with AML and to correlate their expression levels with clinical and Laboratory data of the patients, especially those known to have a prognostic feature.

## Materials and Methods

*Patients and Methods*

This study was carried out on 87 consecutive newly diagnosed adult AML patients, who presented to adult medical oncology department outpatient clinic at the National Cancer Institute, Cairo University over the period of two years. In addition, 20 healthy donors of matched age and sex from donors for bone marrow transplantation were included as a control group. De novo AML was defined if the patient had no history of prior treatment with chemotherapy and had no prior diagnosis of myelodysplastic syndrome or chronic myeloid leukemia. An informed consent was obtained from all the participants prior to the study accompanied by detailed explanation of the procedure and its outcome. This study was carried out in accordance to the guidelines approved by the Ethics Committee, National Cancer Institute, Cairo University; Local institutional research board approval was also acquired prior to the study. The data were collected from patients’ files after permission. 

*Inclusion criteria*

Adult age group (18-70), Egyptian De novo acute myeloid leukemia patient.

*Exclusion criteria*

Pediatric, Non-Egyptians or Treated acute myeloid leukemia patients and Acute promyelocytic leukemia patients.

Patients were analyzed for the following: A: Demographic characteristics including, B: Full history taking including, C: Full clinical examination, D: Laboratory investigations. 

• Bone marrow aspiration and examination, supplemented with cytochemical stains such as Myelo-Peroxidase (MPO) or Sudan Black Stain (SBB), Esterase, Acid Phosphatase and Periodic Acid Schiff (PAS) when indicated. 

• Immunophenotyping for all cases using flow cytometric analysis on routine basis (Using Coulter Epics XL-MCL flow cytometry system). Using leukemia panel detecting myeloid markers (CD13, CD33, CD117, CD14, CD15) in addition to lymphoid markers (CD10, CD19, CD22, CD79a, CD20, Cyto IgM, Kappa and Lambda for B lymphoid series, and CD3, CD2, CD4, CD8, CD7 and CD5 for T lymphoid series) and the stem cell marker CD34 as well as CD56 and HLADR on routine basis. 

• Conventional karyotyping for common cytogenetic abnormalities as t (8:21), inv 16, t (16:16) t (5:17) t (9:12) trisomy 21 and trisomy 3.

• Quantitative analysis of LCN, BCL2L12 genes expression by real time reverse transcriptase polymerase chain reaction (RT-PCR). 

*Assessment of LCN, BCL2L12 expression by real-time (RT-PCR)*

Total RNA was extracted from bone marrow cells using QIAamp RNA extraction blood Mini kit (QIAGEN, cat no. 4440043) as recommended by the manufacturer’s instructions. The purity and the concentration of the extracted RNA were detected using spectrophotometer nano-drop (Quawell, Q-500, Scribner, USA). Retro-transcription (cDNA) was done by using High-Capacity cDNA Reverse Transcription Kit (Applied Biosystems, cat no. 204141) according to the manufacturer’s instructions. The purity and concentration of cDNA were evaluated and then it was stored in – 20 °C till performing quantitative real-time PCR. PHOX2B mRNA expression was quantified using Taqman Universal PCR Master Mix ӀӀ (Applied Biosystems, Foster City, CA, USA,) and Taqman gene Expression Assay for LCN, Syber green gene Expression assay for BCL2L12 (Thermo Fisher Scientific, USA, Hs 00243679). 

RT-PCR was performed using a total volume of 20 μl, and the thermal reaction conditions were as follows: 95°C for 10 min (polymerase activation), followed by 40 cycles of 95°C for 30s (Denaturation), 60°C for 60s (annealing and extension), in which fluorescence was acquired and detected by Step One Real-Time PCR System (Applied Biosystems, Foster City, CA, USA). Relative expression of LCN, BCL2L12 gene were analyzed by the comparative Ct method (2^−ΔΔCt^), in which data were expressed as the fold changes in LCN, BCL2L12 gene expressions in the patients normalized to the expression levels of the endogenous control gene (B Actin, GAPDH) respectively and relative to the healthy controls.

*Statistical Methods*

Data management and analysis were performed using SPSS, (version 20 for Windows; SPSS Inc., Chicago, IL, USA). Categorical data were summarized as percentages; numerical data were summarized using means and standard deviation or medians and ranges. Relation between LCN, BCL2L12 and other variables were assessed using Chi-square test. All tests of hypotheses were conducted at the alpha level of 0.05, with 95% confidence interval. A p-value < 0.05 was considered significant.

## Results

The current study included 87 newly diagnosed adult AML patients with age ranged from 20 to 70 years, out of which,75 patients were evaluated for both LCN and BCL2L12, and all 87 patients were evaluated for LCN, together with twenty age and sex matched healthy controls. Expression levels of LCN, BCL2L12 were determined by real time quantitative reverse transcriptase polymerase chain reaction (RT- q PCR). (Demographic and laboratory characteristics of patients are summarized in Table 1).

The median value of gene expression in control group was taken as a cut off (1.5±0.32 for LCN gene). Below this value the patients are considered as low expressers and above it as high expressers, Seven AML patients (8.2%) had elevated LCN expression and seventy-eight patients (91.3%) had low expression. No statistical significant difference was detected between AML group and control group as regard LCN gene expression levels (p value: 0.48) (Table 2)

**Figure 1:**
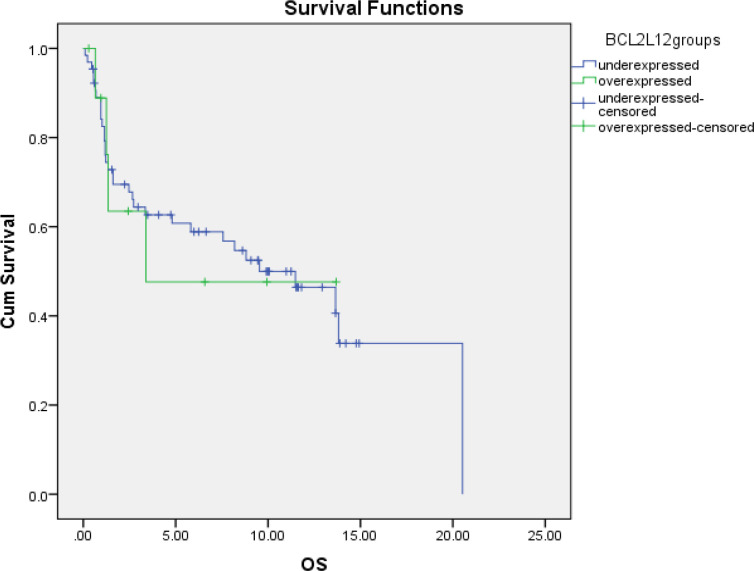
Overall Survival (OS) and LCN Groups in AML patient. OS, Overall Survival; Cum survival, Cumulative Survival

There was statistical significant relation between high and low LCN gene expression in AML patients regarding the sex (P value: 0.01). Comparison revealed no statistical significant relation between high and low LCN gene expression in AML patients regarding demographic and clinical data: age (P value:0.77), hepatomegaly (P value: 0.66), splenomegaly (P value: 0.73) and lymphadenopathy (P value:0.65). Regarding FLT3 there was no statistical significant relation (P value: 0.41) with LCN gene expressers. There was no statistical significant difference between high and low LCN gene expressers AML patients regarding outcome of treatment on day 15 (P value: 0.41), regarding relapse risk (RR) (P value: 0.27) and regarding survival (P value: 0.54, Figure 1).

Regarding BCL2L12 gene expression, we examined 75 patients only (due to inadequate samples). For BCL2L12 gene expression there was no statistical significant difference was detected between AML group and control group as regard BCL2L12 gene expression levels (p value:0.48) (Table3).

There was statistical significant relation between high and low BCL2L12 expressers AML patients regarding the sex (P value: 0.01).

Comparison revealed no statistical significant relation between high and low BCL2L12 gene expression in AML patients regarding demographic and clinical data: age (P value:0.45), hepatomegaly (P value: 0.66) and splenomegaly (P value: 0.73) and lymphadenopathy (P value: 0.65). 

**Figure 2:**
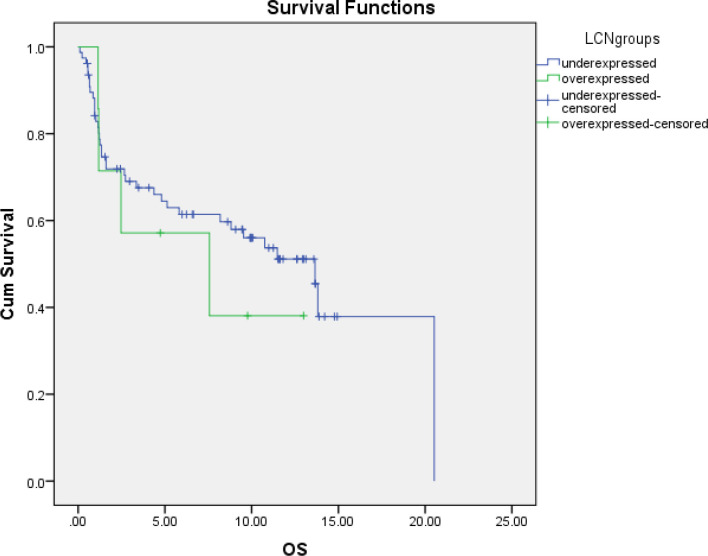
Overall Survival (OS) and *BCL2L12* Groups in AML Patient; OS, Overall Survival; Cum survival, Cumulative Survival

Regarding FLT3 there was no statistical significant relation (P value: 0.41) with BCL2L12 gene expressions. There was no statistical significant difference between high and low BCL2L12 gene expressers AML patients regarding outcome of treatment on day 15 (P value: 1), regarding relapse risk (RR) (P value: 0.1) and survival (P value: 0.94, fig2). There is no significant correlation was observed by the spearman’s correlation test between LCN and BCL2L12 expression.

**Table 1:** Clinico-Pathological Features of the Assessed AML

	Number	Percentage %
Gender		
Male	30	35.3%
Female	55	64.7%
Clinical presentation		
Hepatomegaly	26	30.6%
Splenomegaly	18	21.2%
Lymphadenopathy	19	22.3%
Bone Marrow cellularity		
Normocellular	10	11.8%
Hypercellular	75	88.2%
Cytogenetic abnormalities		
t (8;21)	7	8.2%
Inv 16	4	4.7%
Immunophenotyping		
CD34 Positive	70	83.3%
CD34 Negative	15	17.7%

**Table 2:** Comparison between AML Group and Control Group Regarding the Level of LCN Expression

LCN	AML group N=85 (%)	Control group N=22 (%)	Total	P value
Under expression	78	14	92	0.48
Over expression	7	8	15	

**Table 3:** Comparison between AML Group and Control Group Regarding the Level of BCL2L12

BCL2L12	AML group N=75 (%)	Control group N=20 (%)	Total	P value
Under expression	65	12	77	0.48
Over expression	10	8	18	

## Discussion

AML has a great variability in the pathogenesis. It requires the cooperation between at least two classes of gene mutations (Godley and Shimamura, 2017). 

 Thus, in this study we evaluated the expression of Lipocalin 2 (LCN) and BCL2L12, in newly diagnosed bone marrow samples taken from adult with Acute Myeloid Leukemia and to correlate their expression levels with clinical, laboratory data of the patients and prognosis and patient survival. 

In our study LCN was under expressed in 78 (91.7 %) patients out of 85 adult AML patients, and there were 7 (8.3%) patients have LCN overexpression. There was no significant statistical difference between over expression and under expression of Lipocalin2 in the studied adult AML bone marrow samples compared to the control BM samples as regard outcome of treatment on day 15 (P value: 0.41), or relapse risk (RR) (P value: 0.27) and overall survival (P value :0.54). 

In our study, no statistical significant difference was found between the expression level of LCN2 and age, clinical parameters and hematological parameters (WBC count, hemoglobin, platelets, peripheral blood and bone marrow blasts), immunophenotyping and karyotyping. 

On the contrary to our study, Yang et al 2013 also investigated the expression of LCN2 in a patient’s group and found LCN2 high expression was associated with better prognosis, and FLT3 status had an adjuvant effect on overall survival. 

On the other hand, (Bouch and Bauvois 2014) noticed that NGAL expression in the bone marrow is lower in AML patients than in normal controls. Likewise, NGAL expression increased in patients achieving complete remission and falls in patients with refractory disease. In addition, a combination of FLT3-ID mutation status and high NGAL levels is predictive of the best survival rates in patients with AML. These data suggest that MMP-9 and NGAL might be surrogate markers of disease status in patients with AML. 

Also, study conducted by Bauvois 2018 revealed that the levels of bone marrow NGAL transcript are found to be lower in AML patients than in healthy individuals, and these levels recover to normal values following complete remission and then decline again at relapse.

In patients with AML, higher bone marrow NGAL mRNA expression is observed in individuals with a good prognosis than in individuals with a poor prognosis and independently of the French-American-British (FAB) classification. 

Up to our knowledge, there were no other studies done on LCN2 expression in AML patients and our study is the first study in Egypt. 

However, some studies have reported differentially altered neutrophil gelatinase-associated lipocalin (NGAL) levels in several solid malignancies. They evaluated NGAL measured in plasma or urine as both prognostic and diagnostic marker for different types of human tumor. 

Roli et al., (2017) noticed that positive NGAL expression was associated with a decrease of DFS in patients affected by breast and colorectal cancers, advancing a predictive role of malignancy recurrence.

Likewise, this study revealed a significant association between NGAL overexpression and OS for colorectal and endometrial cancers, suggesting that it could be a useful marker to predict patient survival in these kinds of disease. 

This finding is also coherent with the positive association reported in single studies on liver, lung, esophageal, oral and pancreatic and kidney cancers. In contrast, the study did not find any meaningful association between overexpression of NGAL and OS for gastric cancer. 

In our study there were 65 (86.6 %) patients who have BCL2L12 under expression out of 75 adult AML patients, and 10 (13.4 %) patients have BCL2L12 overexpression. There was no significant statistical difference between over expression and Under expression of BCL2L12 in the studied adult AML bone marrow samples compared to the control BM samples as regard outcome of treatment on day 15 (P value:1), or relapse risk (RR) (P value: 0.1) and overall survival (P value:0.94) 

In our study, no statistical significant difference was found between the expression level of BCL2 and age, clinical parameters and hematological parameters (WBC count, hemoglobin, platelets, peripheral blood and bone marrow blasts), immunophenotyping and karyotyping. 

This result is in agreement with the study done by Zhou et al., (2019), which revealed that BCL2 over expression identified specific FAB subtypes of AML, but it did not affect prognosis.

Also, with subsequent studies done by Kulsoom et al., (2018) which showed that Expression of Bax and Bcl-2 does not differ significantly among AML patients in terms of remission, relapse, resistance, overall survival, and disease-free survival suggest that there is no significant association between the expression of Bax and Bcl-2 and their ratio with clinical response, or with 1-year DFS and OS.

Although both Bax and Bcl-2 had higher expression in those with persistent remission (good response group), a higher Bax/Bcl-2 ratio was more common among those who were resistant or had relapse (poor response group).

In contrast to our study Thomadaki et al., (2012).noticed that Leukemia patients expressing high level of BCL2L12 were 3 times more likely to relapse (p=0.004) or die (p=0.007) than patients with low level of BCL2L12 expression. 

Additionally, statistical significant relationships were found between BCL2L12 expression level and CD117 expression, the presence of splenomegaly and chemotherapy response.

Also, on the contrary to our study, Campos et al., (1993) showed that high expression of BCL2 was associated with a low complete remission rate after intensive chemotherapy, and with a significantly shorter survival. 

In multivariate analysis, the percentage of bcl-2+ cells (or the blast survival in culture), age, and the percentage of CD34+ cells were independently associated with poor survival. 

Up to our knowledge, it is to be noted that our study is the only study to detect both LCN and BCL2L12 expression in AML patients. The discrepancy between our results and other studies may be due to the small sample size and difference in ethnicity, geography, gender and other factors.

In conclusion in our study LCN, BCL2 show no significant difference in the studied adult AML bone marrow samples compared to the control BM samples, but needed to be studied on a large scale of patients. 

## Author Contribution Statement

The contributions of all authors must be described in the following manner: The authors confirm contribution to the paper as follows: study conception and design: X. Author, Y. Author; data collection: Y. Author; analysis and interpretation of results: X. Author, Y. Author. Z. Author; draft manuscript preparation: Y. Author. Z. Author. All authors reviewed the results and approved the final version of the manuscript.
